# Retrospective, observational study of different medication regimens and outcome in children with cough variant asthma

**DOI:** 10.1002/iid3.1357

**Published:** 2024-08-07

**Authors:** Nannan Lou, Xiang Ma, Qingxin Luo, Xiaoling Wei, Yun Zhang, Jing Guo, Jing Wang, Zhongtao Gai

**Affiliations:** ^1^ Department of Respiratory Diseases Children's Hospital Affiliated to Shandong University (Jinan Children's Hospital) Jinan Shandong China; ^2^ Jinan Key Laboratory of Pediatric Respiratory diseases Jinan Children's Hospital Jinan Shandong China; ^3^ Department of Biostatistics, School of Public Health, Cheeloo College of Medicine Shandong University Jinan People's Republic of China; ^4^ Institute for Medical Dataology, Cheeloo College of Medicine Shandong University Jinan People's Republic of China

**Keywords:** children, cough variant asthma, treatment strategy

## Abstract

**Objective:**

This retrospective longitudinal cohort study aimed to explore the best therapeutic regimen and treatment duration of cough variant asthma (CVA) in children.

**Methods:**

A total of 314 children with CVA were divided into receive inhaled corticosteroids (ICS) combined with long‐acting beta2‐agonist (LABA) group, ICS combined with leukotriene receptor antagonists (LTRA) group, ICS monotherapy group and LTRA monotherapy group. All clinical data were statistically analyzed. Logistic regression model was used to compare the advantages and disadvantages of different treatment schemes at each follow‐up time point and the best treatment scheme. The Cox proportional hazard regression model based on inverse probability weighting was used to compare the effects of different medication regimens on adverse outcomes with asthma recurrence or progression as the end point.

**Results:**

(1) After comprehensive analysis, ICS + LABA group was the preferred control regimen for CVA within 8 weeks. After 8 weeks of diagnosis, the efficacy of ICS group or LTRA group was comparable to that of ICS + LABA group and ICS + LTRA group. (2) The ICS + LABA group showed a significant improvement in cough at an early stage, particularly at 4 weeks; the symptoms of ICS + LTRA and ICS groups were significantly improved at 36 weeks. The LTRA group alone showed significant improvement at 20 weeks.

**Conclusion:**

ICS + LABA, ICS + LTRA, ICS alone and LTRA alone can effectively treat CVA. ICS + LABA could improve the symptoms most quickly within 8 weeks after CVA diagnosis, followed by ICS + LATR group. After 8 weeks, it can be reduced to ICS alone to control CVA for at least 36 weeks based on the remission of symptoms in children.

## 1 BACKGROUND

Cough variant asthma (CVA) is a phenotype of asthma with chronic cough as the only symptom, without chest tightness, shortness of breath, or wheezing. The concept of CVA was first proposed by Corrao et al. in 1979.^1^ CVA shares several pathophysiological features with typical asthma (TA), such as airway hyperresponsiveness, chronic airway inflammation, and airway remodeling.^2^, ^3^, ^4^ There are also differences between CVA and TA. For example, chronic inflammation in TA involves the entire lung, whereas CVA is predominantly characterized by inflammation of the small airways without significant damage to the large airways.^5^ In addition, spirometry in patients with CVA is almost normal, with no significant bronchoconstriction or airway obstruction, while TA patients have varying degrees of obstruction in the large airway and small airway.^6^, ^7^, ^8^ CVA is the most common cause of chronic cough,^9^, ^10^, ^11^, ^12^, ^13^ particularly in children, accounting for about 40% of the causes of chronic cough among Chinese children, which greatly affects their learning, sleep, and quality of life.^9^, ^14^ In addition, about 40%–50% of CVA patients may eventually develop wheezing symptoms and progress to TA if no appropriate and timely treatment is given.^15^, ^16^ Over the past decade, the prevalence of asthma in children has been alarmingly increasing annually worldwide.^17^, ^18^ Currently, TA has established a clear and effective staging and grading therapeutic scheme.^19^ However, the treatment plan and duration of children with CVA remain unclear.

Global strategy for asthma management and prevention, guidelines for bronchial asthma prevention and management, and Chinese national guidelines on diagnosis and management of cough considered that the treatment principle of CVA is the same as TA.^19^, ^20^, ^21^ Most patients are effectively treated with inhaled corticosteroids (ICS) or ICS plus long‐acting beta2‐agonist (LABA), and the treatment course is for more than 8 weeks (moderate quality evidence and strongly recommended).^20^, ^21^ Leukotriene receptor antagonists (LTRA) are effective in treating CVA (moderate quality evidence and weakly recommended).^21^ Moreover, LTRA can be used for children who are unable or unwilling to use ICS,^22^ and it can also be considered when CVA with severe airway inflammation or ICS treatment is not effective.^20^ Nevertheless, the preferred medication and treatment duration remain unclear, particularly in children. However, clinical studies on CVA treatment strategies and courses are rare.^21^, ^23^, ^24^ Therefore, in this study, we compared the clinical efficacy and medication duration of ICS alone, LTRA alone, ICS + LTRA, and ICS + LABA to provide a reference for drug selection and course of treatment for CVA.

## 2 MATERIALS AND METHODS

### 2.1 Study population and data collection

A total of 314 children with CVA who were treated and regularly followed up in Children's Hospital Affiliated with Shandong University from October 2019 to June 2022 were included in this retrospective cohort study. These children who were first diagnosed with CVA were divided into four groups based on the initial treatment regimen: (1) ICS + LABA group (group 1): patients were treated with budesonide formoterol or salmeterol fluticasone propionate dry powder; (2) ICS + LTRA group (group 2): were treated with budesonide or fluticasone aerosol inhalation plus oral montelukast; (3) ICS group (group 3): were treated with budesonide or fluticasone aerosol alone; and (4) LTRA group (group 4): were treated with oral montelukast alone. During follow‐up, the treatment plan was up‐ or downregulated based on the condition. The follow‐up time was set according to the guidelines and clinical experience: 2 weeks, 4 weeks, 8 weeks, 12 weeks, 16 weeks, 20 weeks, 28 weeks, 36 weeks, 48 weeks, 60 weeks.^20^ Children with clinical data of 8 weeks or more were included in the study. Information on symptom control, lung function and FeNO results, medication use, whether the medication was discontinued, whether there was a relapse, and whether there was progression to TA was collected at each follow‐up visit for statistical analysis. Symptom control was defined as the absence of daytime and nighttime cough symptoms, the absence of reliever medication use, and the absence of asthma‐induced activity limitation in the past 4 weeks. Disease relapse was defined as the recurrence of an irritating dry cough with the same nature and characteristics of CVA after achieving and maintaining clinical remission for 3 months. Progression to TA was defined as the development of chest tightness, wheezing, and shortness of breath in children with CVA.

### 2.2 Diagnostic criteria for CVA

(1) Chronic cough (lasting more than 4 weeks without specific cause) as the only symptom, the cough was primarily nocturnal and often dry or productive with minimal amounts of clear sputum; (2) no clinical signs of infection, or after a long time (2–4 weeks) ineffective antibiotic treatment; (3) experimental treatment of antiasthma drugs (anti‐inflammatory treatment containing ICS for more than 2 weeks) is effective; (4) other causes of chronic cough were excluded; (5) a positive finding of acetylcholine bronchial provocation test and/or variability in diurnal peak expiratory flow of greater than 13% (continuous monitoring for 2 weeks); and (6) individuals or first and second‐degree relatives have allergic diseases or have tested positive for allergens (the above items 1–4 are the basic conditions for diagnosis).^22^

### 2.3 Inclusion criteria were as follows

(1) children aged 3–14 years old; (2) diagnosed as CVA; (3) adhered to the follow‐up of at least 8 weeks, and the number of follow‐ups ≥ 3 times; and (4) with complete clinical data.

### 2.4 Exclusion criteria were described as follows

(1) children who were not diagnosed in our hospital for the first time; (2) Complicated with sinusitis, chronic obstructive pulmonary diseases, interstitial lung diseases, lung cancer, bronchiectasis and/or other chronic respiratory diseases; (3) Combined with coagulation dysfunction, cardiovascular disease, liver and kidney dysfunction and/or other important organ dysfunction; and (4) combined with pertussis, tuberculosis, *Mycoplasma pneumonia* and/or other infectious diseases in any stage of the treatment.

### 2.5 Criteria of the efficacy evaluation

The evaluation of efficacy was divided into two degrees: controlled and uncontrolled. Control was defined as no cough symptoms, no remission of drug use, and no activity limitation caused by asthma during the day and night in the past 4 weeks. Meanwhile, uncontrolled indicated that in the past 4 weeks, daytime or nighttime cough symptoms >2 times/week, the number of use of relief drugs >2 times/week, and there was limited activity because of asthma.^20^

### 2.6 Auxiliary inspection

#### 2.6.1 Spirometry test

For children under 5 years of age, we used the impulse oscillometry test, while the spirometry test was applied to children over 5 years of age. The indicators and interpretation criteria of pulmonary function were performed based on the current Chinese children's lung function series guidelines.^25^, ^26^

#### 2.6.2 FeNO

The FeNO testing is applicable to children of all age groups. FeNO was measured and interpreted following the American Thoracic Society/European Respiratory Society (ATS/ERS) clinical guidelines.^27^, ^28^, ^29^

### 2.7 Statistical analysis

**Figure 1:**
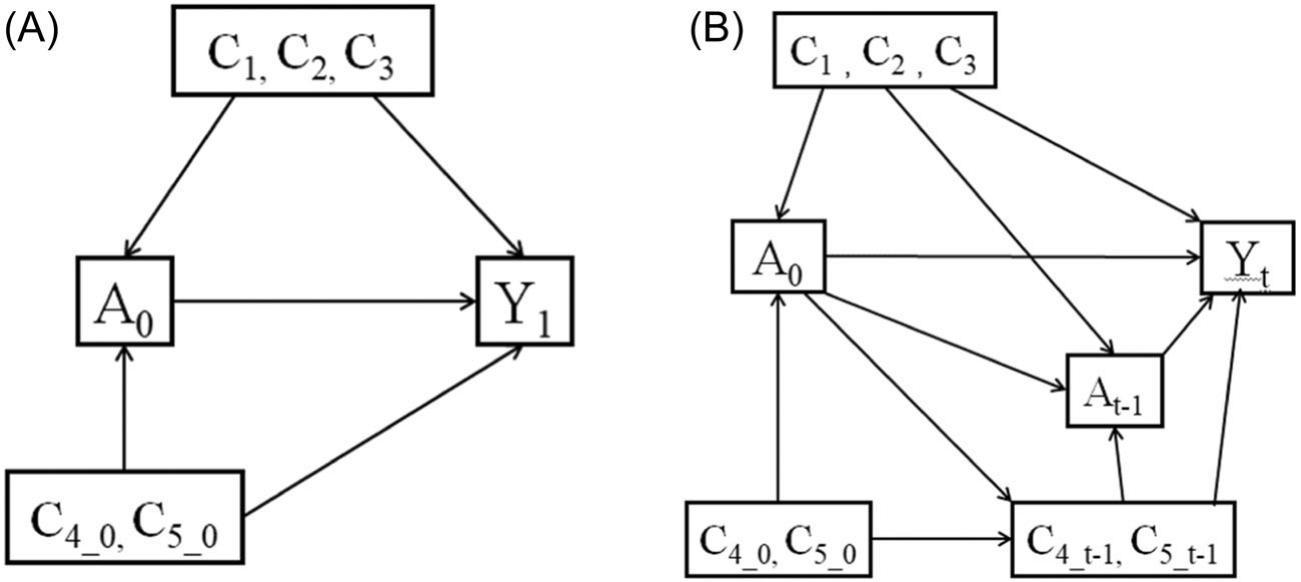
Directed acyclic graph of causality between initial medication regimen and outcome variables. A, medication plan; Y, outcome; *t*, number of follow‐up visits; A_0_, first medication regimen; A_*t*‐1_, medication regimen at *t*–1 follow‐up; Y_1_, outcome at the first follow‐up; Y_*t*_, outcome at *t* follow‐up; Time‐invariant confounding: C_1_, gender, C_2_, age at baseline, C_3_, medical history at baseline; C_4_0_, pulmonary function at first visit; C_4_*t*–1_, Pulmonary function at *t*–1 follow‐up; C_5_0_: fractional exhaled nitric oxide results of the first visit; C_5_*t*–1_: fractional exhaled nitric oxide results at *t*–1 follow‐up; and direction of the arrow: the index of the starting point will affect the index of the arrow pointing.

In this article, we focus on the effect of initial medication regimen (A_0_) on outcome(Y) (Figure 1), and the causal diagrams are designed to show the interaction between other factors included in the study and outcome, including the confounders age (C_1_), sex (C_2_), and medical history (C_3_) that did not change over time, the confounders lung function (C_4_) and Fe NO values (C_5_) that changed over time, and the intermediate variable medication regimen (A_*t*–1_). In view of the fact that lung function (C_4_) and FeNO value (C_5_) have no time regularity in the detection of the previous course of disease, they cannot be analyzed as outcome variables, so they are only used as time‐varying confounding. Logistic regression model was used to compare the efficacy of medication regimens and the optimal course of treatment in each group. Cox proportional hazard regression model was used to compare the effects of different medication regimens on adverse outcomes. The statistical analysis of this study was performed using the software package R 4.1.2. The post hoc comparison Bonferroni method was chosen for the statistical analysis process. The data were expressed as mean ± SD or *n* (%). The odds ratio (OR) and hazard ratio (HR) were 1 as a positive event, that is, the closer to 0, the better the protective effect. *p* < .05 indicated that the difference was statistically significant.

#### 2.7.1 Superiority analysis

##### Comparison of therapeutic effect of drugs

Based on the causal diagram (Figure 1) model assumption, groups 2, 3, and 4 were compared with group 1 as the control group with clinical control as the outcome. Given that the observation data in this study are repeated measurement data, the medication regimen and some covariates will change with time. Therefore, the marginal structure model is fitted to balance the confounding factors between groups and control the impact of subsequent medication changes on the outcome to obtain the direct causal effect of the first medication on each outcome and compare the advantages and disadvantages of different treatment regimens at each follow‐up time point.

##### Study on the optimal course of medication

Based on the causal diagram (Figure 1) on exposure (medication plan) and outcome (clinical control), group 1 was compared with groups 2, 3, and 4 (nongroup 1), and the same. The marginal structure model was fitted to obtain the direct causal effect of each group on the outcome of each follow‐up. Then, the differences in the effectiveness of different treatment regimens at different follow‐up time points were compared with understand the optimal course of treatment for each group.

#### 2.7.2 Adverse outcome analysis

Herein, groups 2, 3, and 4 are compared with group 1 with the occurrence of recurrence or progression to TA as the adverse outcome. The Cox proportional hazards regression model is used to correct the influence of confounding factors and control the change of medication regimen, estimate the direct causal effect of the first medication on adverse outcomes and compare the occurrence of adverse outcomes based on inverse probability weighting.

## 3 RESULTS

### 3.1 Basic information of enrolled patients

No significant difference was observed in gender and FeNO between the four groups (*p* > .05). However, significant differences were found in the average age of patients, medical history, and pulmonary function among the four groups (*p* < .05). In this study, there are some differences in the baseline data of patients because of the large age span of children and the different means of pulmonary function testing. Given this, the marginal structure and Cox proportional hazard regression models were used to adjust for the confounding factors such as age, gender, medical history, lung function, and FeNO based on inverse probability weighting so that the four groups of data in this study were balanced and comparable (Table 1).

**Table 1:** General Information of 314 Children with CVA.

Characteristics	Level	ICS + LABA (group 1)	ICS + LTRA (group 2)	ICS (group 3)	LTRA (group 4)	*p* Value
Number of patients (*n*)		63	186	30	35	
Gender (*n*, %)	Females	14 (22.2)	62 (33.5)	11 (36.7)	14 (40.0)	.234
	Males	49 (77.8)	123 (66.5)	19 (63.3)	21 (60.0)	
Age (years, mean ± SD)		7.75 ± 1.75	5.39 ± 1.54	5.51 ± 1.72	5.27 ± 2.01	<.001
Cough duration (months, mean ± SD)		8.32 ± 13.79	4.21 ± 7.66	3.17 ± 3.09	3.06 ± 4.32	.004
Lung function (*n*, %)	Normal	57 (95.0)	126 (77.3)	19 (73.1)	20 (80.0)	.002
	Abnormal	0 (0.0)	35 (21.5)	5 (19.2)	5 (20.0)	
	Small airway anomaly	3 (5.0)	2 (1.2)	2 (7.7)	0 (0.0)	
FeNO (*n*, %)	Normal	33 (60.0)	71 (52.2)	11 (61.1)	14 (63.6)	.602
	Abnormal	22 (40.0)	65 (47.8)	7 (38.9)	8 (36.4)	

*Note*: Data are expressed as mean ± standard deviation or quantity (percentage); *p* < .05 was considered statistically significant.

Abbreviations: CVA, cough variant asthma; FeNO, fractional exhaled nitric oxide; ICS, inhaled corticosteroids; LABA, ICS + long‐acting bete2‐agonist; LTRA, leukotriene receptor antagonists.

### 3.2 Quantitative distribution

**Figure 2:**
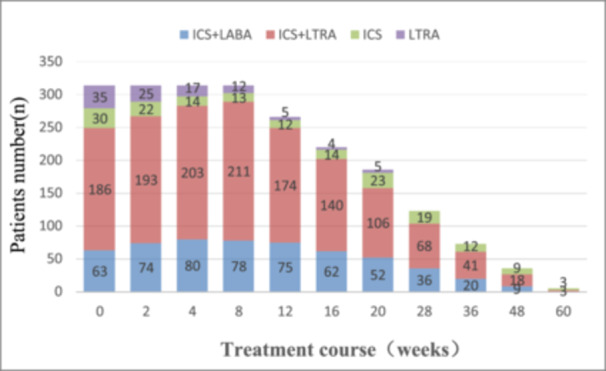
The number distribution of patients with four treatment regimens under different duration of medication. ICS, inhaled corticosteroids; LABA, ICS + long‐acting bete2‐agonist; LTRA, leukotriene receptor antagonists.

A total of 314 patients persisted in follow‐up and treatment for more than 8 weeks, and subsequently, some patients gradually fell out. For example, after the 8th week, 37 people were transferred to local hospitals for further treatment, two people stopped taking medication on their own, and nine people lost contact. Therefore, during the 12th‐week follow‐up, 260 people continued to be treated at our hospital. When followed up to week 60, five people were still receiving treatment in our hospital. However, when there are only a few children followed up to the 48th and 60th week of medication, it is not included in the final data analysis (Figure 2).

### 3.3 Results of superiority analysis (with group 1 as the control)

Based on the clinical guidelines,^20^, ^21^ the follow‐up was divided into two parts: within 8 weeks and after 8 weeks. The efficacy of the four regimens within 8 weeks of treatment was compared (Table 2): Groups 2, 3, and 4 were different in controlling the risk of cough symptoms in CVA children as compared with group 1, however, the differences were not statistically significant (95% confidence interval of odds ratio [OR] values included 1, and *p* > .05).

**Table 2:** Comparison of the efficacy of different medication duration in 314 children with CVA (within 8 weeks).

treatment duration	Characteristics	*β*	SE	OR (95% CI)	Wald	*p* Value
2 weeks	ICS + LTRA versus ICS + LABA	0.014	0.071	1.014 (0.881–1.166)	0.036	.849
	ICS versus ICS + LABA	0.078	0.117	1.081 (0.859–1.360)	0.441	.506
	LTRA versus ICS + LABA	0.095	0.088	1.100 (0.926–1.306)	1.174	.279
4 weeks	ICS + LTRA versus ICS + LABA	0.097	0.115	1.102 (0.879–1.382)	0.709	.400
	ICS versus ICS + LABA	0.224	0.161	1.251 (0.913–1.715)	1.948	.163
	LTRA versus ICS + LABA	0.020	0.158	1.020 (0.748–1.391)	0.016	.899
8 weeks	ICS + LTRA versus ICS + LABA	0.005	0.082	1.005 (0.856‐–1.181)	0.004	.947
	ICS versus ICS + LABA	− 0.018	0.113	0.982 (0.787–1.225)	0.026	.871
	LTRA versus ICS + LABA	0.143	0.124	1.154 (0.905–1.471)	1.331	.249

*Note*: *p* < .05 was considered statistically significant.

Abbreviations: CI, confidence interval; ICS, inhaled corticosteroids; LABA, ICS + long‐acting bete2‐agonist; LTRA, leukotriene receptor antagonists; OR, odds ratio; SE, standard error.

Comparison of the efficacy of the four treatment regimens after 8 weeks of treatment (Table 3): The four treatment regimens began to show significant differences after 8 weeks as compared with within 8 weeks. A difference was observed in the risk of cough symptom control in children with CVA in group 2 as compared with group 1, however, the difference was not statistically significant (*p* > .05). Meanwhile, groups 3 and 4 reduced the risk of uncontrolled CVA from week 12 to week 36 as compared with group 1, and the difference was statistically significant (OR < 1, *p* < .05).

**Table 3:** Effect of treatment duration on the therapeutic effect of CVA children (>8 weeks).

Treatment duration	characteristics	*β*	SE	OR (95%CI)	Wald	*p* Value
12 weeks	ICS + LTRA versus ICS + LABA	− 0.025	0.056	0.975 (0.874–1.088)	0.199	.656
	ICS versus ICS + LABA	− 0.162	0.038	0.850 (0.789–0.916)	18.288	.000
	LTRA versus ICS + LABA	− 0.080	0.036	0.923 (0.860–0.992)	4.780	.029
16 weeks	ICS + LTRA versus ICS + LABA	0.005	0.066	1.005 (0.882–1.144)	0.005	.943
	ICS versus ICS + LABA	− 0.236	0.042	0.790 (0.727–0.859)	30.757	.000
	LTRA versus ICS + LABA	− 0.126	0.055	0.882 (0.793–0.981)	5.317	.021
20 weeks	ICS + LTRA versus ICS + LABA	0.025	0.062	1.025 (0.907–1.158)	0.159	.690
	ICS versus ICS + LABA	− 0.182	0.041	0.834 (0.770–0.903)	19.901	.000
	LTRA versus ICS + LABA	− 0.164	0.038	0.848 (0.788–0.914)	18.890	.000
28 weeks	ICS + LTRA versus ICS + LABA	0.038	0.043	1.039 (0.955–1.130)	0.791	.374
	ICS versus ICS + LABA	−0.151	0.043	0.860 (0.791–0.935)	12.521	.000
	LTRA versus ICS + LABA	−0.092	0.038	0.912 (0.846–0.983)	5.849	.016
36 weeks	ICS + LTRA versus ICS + LABA	−0.062	0.047	0.940 (0.857–1.031)	1.708	.191
	ICS versus ICS + LABA	−0.300	0.145	0.741 (0.557–0.985)	4.253	.039
	LTRA versus ICS + LABA	−0.099	0.049	0.906 (0.823–0.997)	4.062	.044

*Note*: *p* < .05 was considered statistically significant.

Abbreviations: CI, confidence interval; ICS, inhaled corticosteroids; LABA, ICS + long‐acting bete2‐agonist; LTRA, Leukotriene receptor antagonists; OR, odds ratio; SE, standard error.

Comparison of therapeutic effects at different courses (Table 4): ICS + LABA had the best effect within 8 weeks, followed by ICS + LTRA, whereas ICS alone and LTRA alone had the worst efficacy. With the extension of medication time, the efficacy of the ICS + LABA group was gradually lower than other groups in the same period. ICS alone was predominant at 8 weeks and beyond, followed by LTRA alone, whereas ICS + LABA and ICS + LTRA had the worst efficacy.

**Table 4:** Comparison of four medication regimens for CVA children at different courses.

Treatment duration (study population)	Efficacy ranking
2 weeks (314)	ICS + LABA＞ICS + LTRA＞ICS＞LTRA
4 weeks (314)	ICS + LABA＞LTRA＞ICS + LTRA＞ICS
8 weeks (314)	ICS＞ICS + LABA＞ICS + LTRA＞LTRA
12 weeks (266)	ICS＞LTRA＞ICS + LTRA＞ICS + LABA
16 weeks (220)	ICS＞LTRA＞ICS + LABA＞ICS + LTRA
20 weeks (186)	ICS＞LTRA＞ICS + LABA＞ICS + LTRA
28 weeks (123)	ICS＞LTRA＞ICS + LABA＞ICS + LTRA
36 weeks (73)	ICS＞LTRA＞ICS + LTRA＞ICS + LABA

Abbreviations: ICS, inhaled corticosteroids; LABA, ICS + long‐acting bete2‐agonist; LTRA, leukotriene receptor antagonists.

**Figure 3:**
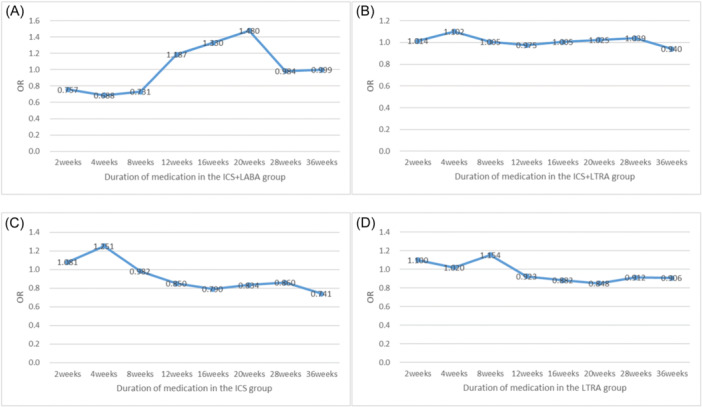
Effective changed from 2 weeks to 36 weeks of ICS + LABA group (A), ICS + LTRA group (B), ICS alone (C), and LTRA alone (D). ICS: inhaled corticosteroids; LABA, ICS+ long‐acting bete2‐agonist; LTRA, leukotriene receptor antagonists.

Results of optimal course comparison with other groups not using the drug as controls (Figure 3): The ICS + LABA group showed a significant improvement in cough at an early stage, particularly in the 4th week, the symptoms significantly improved; meanwhile, the symptoms of ICS + LTRA and ICS groups were significantly improved at 36 weeks. LTRA alone showed significant improvement at 20 weeks.

The adverse outcomes analysis results with group 1 as the control (Table 5): The effect of the LTRA group was the most significant, followed by the ICS and ICS + LABA groups, and then the ICS + LTRA group. The ability of group 2 to reduce the risk of CVA recurrence and progression to TA was slightly worse as compared with group 1, and the difference was statistically significant (HR = 4.045, 95% CI: 1.183–13.831, *p* < .05). In addition, the risk of CVA recurrence and progression to TA was different in group 3 as compared with group 1, however, the difference was not statistically significant (HR = 0.853, 95% CI: 0.198–3.675, *p* > .05). Moreover, group 4 reduced the risk of CVA recurrence and progression to TA as compared with group 1, and the difference was statistically significant (HR = 0.265, 95% CI 0.079–0.894, *p* < .05).

**Table 5:** Comparison of the effects of four different treatment regimens on adverse outcomes.

Characteristics	*β*	SE	HR (95%CI)	*z* Value	*p V*alue	*p* zph
ICS + LTRA versus ICS + LABA	1.398	0.686	4.045 (1.183–13.831)	2.228	.026	0.726
ICS versus ICS + LABA	−0.159	0.692	0.853 (0.198–3.675)	−0.213	.831	0.054
LTRA versus ICS + LABA	−1.327	0.784	0.265 (0.079–0.894)	−2.140	.032	0.622

*Note*: *p* < .05 was considered statistically significant.

Abbreviations: CI, confidence interval; HR, hazard ratio; ICS, inhaled corticosteroids; LABA, ICS+ long‐acting bete2‐agonist; LTRA, leukotriene receptor antagonists; p‐zph, proportional risk assumption test; SE, standard error; z‐value, test statistic.

## 4 DISCUSSION

At present, the best treatment therapy and the best course of treatment for CVA particularly in children is not very clear, thus, a large number of clinical studies are still needed to provide relevant evidence. In this study, four common schemes composed of three drugs commonly used in the treatment of CVA in children were included in the study to explore in depth the drug selection and optimal course of CVA. Hence, we found that the four treatment regimens for CVA children are effective. Furthermore, our study confirms that ICS + LABA is the first choice within 8 weeks of diagnosis; meanwhile, after 8 weeks, ICS alone was more effective, and the cough symptoms improved most significantly 36 weeks after treatment.

Herein, we first investigated and compared the efficacy of four regimens in the treatment of CVA. We found that ICS alone, LTRA alone, ICS + LABA, and ICS + LTRA can effectively relieve cough symptoms in children with CVA, which is similar to previous studies.^30^, ^31^, ^32^, ^33^, ^34^, ^35^, ^36^, ^37^, ^38^, ^39^, ^40^, ^41^ Most of the guidelines recommend that the CVA treatment is for at least 8 weeks, therefore, we divided it into two parts with 8 weeks as the boundary.^20^, ^21^ Our study showed that the therapeutic effects of the four treatment regimens were comparable within 8 weeks of diagnosis of CVA, and no significant difference was observed in cough symptom control among the four groups. According to Fang et al., montelukast alone showed antitussive and anti‐inflammatory effects similar to ICS + LABA or ICS + LABA combined with montelukast.^42^ A survey by Zhu et al. also showed that montelukast alone and fluticasone propionate alone have no significant difference in the treatment of CVA 2 months cough score.^15^ There are also different opinions. A meta‐analysis involving 11 randomized controlled trials showed that in the Chinese population, the efficacy of montelukast sodium combined with budesonide was significantly increased as compared with budesonide alone in the treatment of CVA in children, and did not increase the incidence of related adverse drug reactions.^43^

The results of this study showed that the efficacy of ICS or LTRA alone was no less than that of combination therapy after 8 weeks of diagnosis, thus suggesting that monotherapy could be considered in the subsequent treatment. This situation may be related to the following reasons: (1) After 8 weeks of diagnosis, there were two sources of children in the combined treatment groups: the combined treatment groups with poor symptom control still maintains the combined treatment, and the single drug treatment groups with incomplete symptom control chooses to increase the treatment drugs to the combined treatment groups; (2) There were also two sources of children in the monotherapy groups: the combination therapy is reduced to the monotherapy groups due to good symptom control, and the monotherapy groups with good symptom control continue to maintain the current treatment. In general, it is acceptable to use single drug therapy to control the disease in the late stage of symptom relief, thus suggesting that CVA can be controlled by single drug therapy after 8 weeks of treatment according to the remission of symptoms. A recent study showed that the combination of fluticasone and montelukast in the treatment of CVA in children for 2 months, the relief of cough symptoms, and the improvement of lung function were significantly better than those of single drug use.^15^ However, after 3 months of treatment, the efficacy of fluticasone alone or montelukast alone was not worse than that of combined treatment.^15^

This study also shows that ICS alone is superior to LTRA alone in the single drug. Meanwhile, the ICS + LABA group showed significant improvement in cough in the early stages, the symptoms of the ICS + LTRA and ICS groups significantly improved at 36 weeks of medication, and the LTRA group alone showed significant improvement at 20 weeks. Previous studies have shown that cough symptoms have been controlled in most ICS + LABA‐treated patients or combined with LTRA, however, sputum eosinophilia continued to persist, thus indicating that 8 weeks of treatment are far from enough for improving airway inflammation in CVA patients.^42^ In CVA patients, ICS treatment alone for 3 months, 6 months, and 12 months significantly reduced airway hyperresponsiveness, decreased the proportion of eosinophils in sputum, and improved the levels of interleukin‐5 (IL‐5) and IL‐10 in induced sputum, respectively, thereby suggesting that long‐term ICS treatment remained essential to completely eliminate airway inflammation.^31^

Various scholars believe that long‐term use of ICS treatment can prevent CVA from transforming into TA.^6^, ^32^, ^44^, ^45^, ^46^ In children, the proportion of untreated CVA developing into TA is as high as 54%.^47^ The data of this study showed that all four treatment regimens could reduce the risk of CVA recurrence and progression to TA. The effect of the LTRA group was the most significant, followed by the ICS and ICS + LABA groups, and then the ICS + LTRA group. A randomized controlled study of 112 children with CVA showed that the recurrence rate of children with CVA treated with ICS + LTRA for 8 weeks was similar to that of ICS alone, however, the recurrence rate of ICS + LTRA group was lower than that of the ICS group after 1 year of follow‐up.^48^ Another survey showed that the probability of wheezing with ICS alone is significantly lower than with montelukast alone.^45^ In this case, the sample size of the ICS + LTRA group in this study is quite different from that of the other groups, and the follow‐up time of each group is not equal. There may be unmeasured or unknown confounding, which may have a certain effect on the statistical results.

As far as we know, this study is the first clinical study report of CVA children covering the comparison of four commonly used treatment regimens, with a large total sample size and relatively long follow‐up time, which is of great significance as a treatment guide in treating CVA in children. However, this study also has some limitations. First, this is a retrospective study and the number of people in each group is unevenly distributed. With the prolongation of medication time, the number of children who adhere to treatment and follow‐up gradually decreased. Second, in the absence of specific guidelines, the treatment is based on the physician's personal experience to some extent. In addition, lung function results varied because of children's age and cognitive problems, thereby resulting in related issues difficult to analyze. Therefore, we are currently conducting a prospective survey to obtain better data to guide clinical practice.

## 5 CONCLUSION

The results of our study suggest that all four treatment regimens are effective in children with CVA and that a minimum of 36 weeks of therapy is required. However, the basis for the selection of the medication regimen and the criteria for discontinuation of the medication need to be further investigated.

## AUTHOR CONTRIBUTIONS

**Nannan Lou**: Data curation; investigation; validation; writing—original draft; writing—review & editing. **Xiang Ma**: Funding acquisition; project administration; writing—review & editing. **Qingxin Luo**: Formal analysis; methodology. **Xiaoling Wei**: Funding acquisition; resources; supervision. **Yun Zhang**: Funding acquisition; resources; supervision. **Jing Guo**: Investigation; supervision. **Jing Wang**: Investigation; resources; supervision. **Zhongtao Gai**: Conceptualization; project administration; supervision.

## CONFLICT OF INTEREST STATEMENT

The authors declare no conflict of interest.

## ETHICS STATEMENT

This study passed the medical ethics review of the Children's Hospital Affiliated with Shandong University, and as it was a retrospective study, the requirement for informed consent was waived. We have reviewed the final version of the manuscript and approved it for publication. The study was approved by the Ethics Committee of Children's Hospital Affiliated to Shandong University (The ethics protocol number is SDFE–IRB/T–2022075). All personal identification data were anonymized and deidentified before conducting the analysis.

## Data Availability

All data generated or analyzed during this study are included in this article.
